# Ultrasensitive SERS-Based Plasmonic Sensor with Analyte Enrichment System Produced by Direct Laser Writing

**DOI:** 10.3390/nano10010049

**Published:** 2019-12-24

**Authors:** Georgii Pavliuk, Dmitrii Pavlov, Eugeny Mitsai, Oleg Vitrik, Aleksandr Mironenko, Alexander Zakharenko, Sergei A. Kulinich, Saulius Juodkazis, Svetlana Bratskaya, Alexey Zhizhchenko, Aleksandr Kuchmizhak

**Affiliations:** 1 Far Eastern Federal University, 690041 Vladivostok, Russia; georgii.23542@gmail.com (G.P.); pavlov_dim@mail.ru (D.P.); oleg_vitrik@mail.ru (O.V.); rarf@yandex.ru (A.Z.); skulinich@tokai-u.jp (S.A.K.); g89leksig@mail.ru (A.Z.); 2 Institute of Automation and Control Processes, Far Eastern Branch, Russian Academy of Sciences, 690091 Vladivostok, Russia; eugenemitsai@gmail.com; 3 Institute of Chemistry, Far Eastern Branch, Russian Academy of Sciences, 690091 Vladivostok, Russia; almironenko@gmail.com (A.M.); s.bratskaya@gmail.com (S.B.); 4 Research Institute of Science and Technology, Tokai University, Hiratsuka, Kanagawa 259-1292, Japan; 5 Nanotechnology Facility, Swinburne University of Technology, John st., Hawthorn, VIC 3122, Australia; saulius.juodkazis@gmail.com; 6 World Research Hub Initiative (WRHI), School of Materials and Chemical Technology, Tokyo Institute of Technology, 2-12-1, Ookayama, Meguro-ku, Tokyo 152-8550, Japan

**Keywords:** direct laser processing, femtosecond laser pulses, superhydrophobic textures, analyte enrichment, plasmonic nanostructures, SERS, medical drugs

## Abstract

We report an easy-to-implement device for surface-enhanced Raman scattering (SERS)-based detection of various analytes dissolved in water droplets at trace concentrations. The device combines an analyte-enrichment system and SERS-active sensor site, both produced via inexpensive and high-performance direct femtosecond (fs)-laser printing. Fabricated on a surface of water-repellent polytetrafluoroethylene substrate as an arrangement of micropillars, the analyte-enrichment system supports evaporating water droplet in the Cassie–Baxter superhydrophobic state, thus ensuring delivery of the dissolved analyte molecules towards the hydrophilic SERS-active site. The efficient pre-concentration of the analyte onto the sensor site based on densely arranged spiky plasmonic nanotextures results in its subsequent label-free identification by means of SERS spectroscopy. Using the proposed device, we demonstrate reliable SERS-based fingerprinting of various analytes, including common organic dyes and medical drugs at ppb concentrations. The proposed device is believed to find applications in various areas, including label-free environmental monitoring, medical diagnostics, and forensics.

## 1. Introduction

Surface-enhanced Raman scattering (SERS) is a non-invasive ultrasensitive method permitting the identification of various molecular species via their unique vibrational fingerprints that appear in the measured scattering spectra [1]. Although the probability of inelastic Raman scattering is intrinsically very low, this process can be boosted enormously when the analyzed molecules are attached to nanostructures that support enhanced electromagnetic (EM) near-field “hot spots”. Typically, arrangements of noble-metal nanostructures supporting collective oscillations of free electron plasma, surface plasmons (SPs), are actively used as SERS-active substrates to generate EM hot spots [2,3,4]. Alternatively, all-dielectric nanostructures, as well as hybrid platforms combining plasmonic and dielectric nanostructures with 0D/1D materials, have recently emerged as a promising route towards efficient SERS-active substrates with extended functionalities [5,6,7]. For molecules attached to hot spot-supporting nanostructure, the SERS intensity was shown roughly to scale with a forth power of local EM-field amplitude [8]. Accordingly, achieving the maximum enhancement and local EM-field with carefully designed plasmonic nanostructures (or so-called “hot spot” engineering) is a mainstream direction towards SERS-active substrates with single-molecule detection performance.

Along with EM-mediated SERS enhancement, interaction of the analyte with nanostructures through electron exchange can also compliment the overall SERS yield with an extra chemical enhancement factor, whose contribution ranges from 10 to 100 and depends on the molecule–nanostructure affinity [4,9]. However, to make both of these enhancement mechanisms work efficiently, the analyte molecules should be placed in the closest proximity to the nanostructure, which is very far from being trivial when the analyte is deposited from very dilute solutions [10,11]. This problem cannot be solved with "hot spot" engineering alone, which is why several efficient strategies have been proposed, aiming at targeted delivery of analyte molecules towards SERS-active sites [4]. First of all, specific chemical interactions between analyte molecules and nanoantennas can be used to guide analyte towards EM “hot spots”. Within this approach, one utilizes either chemical modification of the analyte molecules (to enlarge their intrinsically weak Raman cross-section) [12] or intermediate molecules with good affinity to SERS-active nanostructure (to capture selectively the analyte via specific chemical bonds) [13,14]. However, such chemistry-based enrichment strategies are analyte-specific and cannot be considered as a universal tool.

Alternatively, efficient analyte enrichment can be realized via a careful control of wetting behavior of the evaporating water droplet loaded with an analyte. More specifically, non-wetting superhydrophobic surfaces are known to support deposited water droplets in the Cassie–Baxter state, which provides a large water contact angle (CA) and extremely small size of the solid–liquid contact area. Under such conditions, while the droplet evaporates and shrinks, the dissolved analyte molecules can be concentrated and deposited on a small surface area near the SERS-active site [15]. Previously, the majority of developed devices that utilize this analyte-enrichment strategy were produced via expensive and time-consuming lithography-based approaches [15,16], which substantially limited their applicability for routine SERS measurements requiring single-use handling to preserve reliability. Moreover, an analyte-enrichment system was also proposed to form a plasmon-active site by concentrating the chemically synthesized colloids dispersed in the drying droplet. These agglomerated colloids were further used as SERS-active site for analyte identification [15,17]. However, as the SERS performance achieved in the experiments reaches a single-molecule detection level, this approach requires rigorous and intricate purification of the colloids after their synthesis to provide background-free reliable detection of the analyte.

Direct laser processing of various materials with nano- and femtosecond (fs) laser pulses is known to be a facile and inexpensive technology for fabrication of both plasmon-active nanostructures [18,19] and topographies permitting to control surface wetting [20,21,22,23,24,25,26,27]. However, no attempts were made so far to utilize direct laser processing to create a fully functional device that would combine efficient analyte enrichment with ultrasensitive SERS-based detection.

Here, we report an easy-to-implement device for SERS-based identification of various analytes dissolved in water droplets at trace concentrations. The device combines an analyte enrichment system and a SERS-active sensor site, both produced via inexpensive and highly performing direct fs-laser printing. The analyte-enrichment system fabricated on a surface of water-repellent PTFE substrate as an arrangement of micropillars supports the drying water droplet in the Cassie–Baxter superhydrophobic state, thus allowing for delivery of the dissolved analyte towards the hydrophilic SERS-active site. Efficient pre-concentration of the analyte on the sensor site with densely arranged spiky plasmonic nanotextures provides its subsequent label-free identification using SERS spectroscopy. Using the proposed device, we demonstrate reliable SERS-based fingerprinting of various analytes, including common organic dyes and medical drugs at concentrations ranging from 10${}^{-8}$ to 10${}^{-12}$ M.

## 2. Materials and Methods

### 2.1. Fabrication of Analyte-Enrichment Structures and Plasmonic Sensor Element

Laser processing was performed using 180-fs second-harmonic (515 nm) laser pulses generated by a regenerative amplified ytterbium-doped potassium gadolinium tungstate (Yb:KGW) fs-laser system (Pharos, Light Conversion Ltd., Vilnius, Lithuania). First, fs-laser radiation was used to fabricate a device for analyte enrichment, which was recorded on a smooth mechanically polished PTFE surface following the procedure and design previously developed and optimized in [25]. Laser pulses generated at repetition rate 10 KHz were focused on a PTFE surface using a dry microscope objective with numerical aperture (NA) of 0.15, while the sample was arranged on a PC-driven nanopositioning platform (Aerotech Gmbh., Nurnberg, Germany) that permitted accurate laser beam scanning of its surface according to predefined template. Deposition of 600-nm thick Ag and 25-nm thick Au films onto the central site of the sample was performed using e-beam evaporation through a shadow mask (Kurt J. Lesker Co., Jefferson Hills, USA). The same fs-laser setup was used to produce spallative surface textures on a Ag-coated central site. To maximize plasmonic performance of the sensor element, fabrication of spallative textures was carried out using a double pulse processing of the Ag-coated central site (see details in Results and Discussion section). Flat-top circular-shaped laser beam was used to ensure the densest hexagonal arrangement of the spallative textures on the central site. This intensity pattern was generated by guiding the output laser beam through the circular pinhole followed by focusing the output field onto the sample surface with a 4f-focal system comprised of a focusing lens and a dry microscope objective with NA = 0.8.

### 2.2. Device Characterization

Surface morphology of the as-produced device was characterized with scanning electron microscopy (SEM; Ultra 55+, Carl Zeiss, Oberkochen, Germany). Wetting and analyte-concentrating properties of the device were studied using a home-built optical system that allowed for tracking water droplets (5 μL) evaporating and drying on the sample surface. The system captured both top- and side-view images of the droplet each 10 s. To deposit the droplet onto the device surface, we used custom-made hydrophobized quartz capillaries with an output diameter of ≈50 μm. All experiments were performed at 25 ${}^{\circ }$C and relative humidity of 30%. The droplet contour was used then to calculate the drop volume V and CA using standard techniques [28]. More detailed information regarding evaluation of devices wetting characteristics can be found elsewhere [25]. Correlated reflection and dark-field (DF) back-scattering spectra were used to characterize plasmonic performance of the Au-coated spallative textures. Such spectra were measured using a home-built optical microscope confocally aligned with a sensitive spectrometer (Shamrock 303i, Andor Technology, Belfast, Northern Ireland) equipped with a TE-cooled CCD-camera (Newton 971, Andor Technology, Belfast, Northern Ireland). The spectra were acquired with a 0.8-NA dry objective ensuring wide collection angle, while an adjustable pinhole permitted controlling acquisition area size. The s-polarized white-light radiation from a stabilized tungsten bulb (HL2000-HP, Ocean Optics, Largo, USA) was used to provide DF side illumination (at 80${}^{\circ }$ with respect to the sample normal) of the textured surfaces during spectroscopic measurements.

### 2.3. Evaluation of SERS Performance

For the majority of SERS experiments, we used a commercial Raman microscope (Morphologi G3-ID, Malvern Instruments Ltd., Malvern, UK) equipped with a spectrometer (RamanRnx1, Kaiser Optical Systems Inc., Ann Arbor, USA) and a CW laser source with a central wavelength of 785 nm. Output laser radiation was focused on the SERS-active plasmonic site of the sensor through a dry microscope objective with NA = 0.6 (Nikon, 50x TU Plan ELWD, Tokyo, Japan) yielding ≈1 μm${}^{2}$ in its focal spot area. Additionally, to demonstrate the broadband plasmonic response of the sensor, we used two Raman microscopes (Alpha 300, WiTec GmbH, Ulm, Germany and LabRam800 HR, Horiba, Kyoto, Japan) equipped with several laser sources with their wavelength centered at 488, 532, and 632 nm. In all such experiments, the laser fluence onto the sample surface was controlled by an optical power meter to be 0.9 mW/μm${}^{2}$.

For the proof-of-concept demonstration of spectrally broadband SERS performance of the device, Rhodamine 6G (R6G) organic dye was used as a model analyte. This analyte is known to have a good affinity to plasmon-active metals, as well as a large Raman cross-section. Also, 4${}^{\prime }$,6-diamidino-2-phenylindole dihydrochloride (DAPI) fluorescent marker was used for SERS experiments to study the Raman yield as a function of analyte concentration. Moreover, the developed sensor was tested for SERS detection of several widely used biologically active compounds: (1) Pefloxacin; (2) Ciprofloxacin; (3) Levofloxacin (a quinolone antibiotic used to treat numerous bacterial infections); (4) Diphenhydramine hydrochloride (an antihistamine mainly used to treat allergies); (5) Ibuprofen; (6) Diclofenac (a nonsteroidal anti-inflammatory drug used for treating pain, fever, and inflammation); and (7) 4-Acetamidophenol, also known as paracetamol or acetaminophen (a medication used to treat pain and fever).

All the analytes were deposited onto the sensors plasmonic site from water droplets (5 μL in volume) that contained their certain initial concentration (10${}^{-10}$ M for R6G; 10${}^{-9}$, 10${}^{-10}$, 10${}^{-11}$ for DAPI; and 10${}^{-8}$ M for the other analytes) and according to the procedure described in the previous section. Each SERS spectrum discussed below was averaged over 50 similar independent spectra obtained at various locations on the central site of the sensor.

## 3. Results and Discussion

### 3.1. Device Fabrication

**Figure 1:**
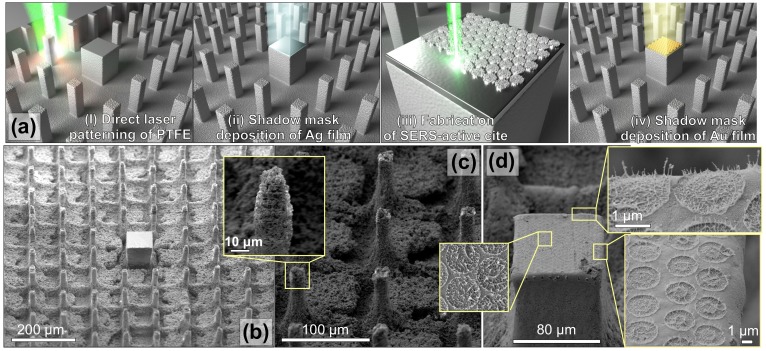
Fabrication of plasmonic sensor with analyte-enrichment system. (**a**) Scheme of the sensor fabrication process: (**i**) direct laser writing of analyte-enrichment system on PTFE surface; (**ii**) deposition of 600-nm thick Ag film onto a central pillar through a shadow mask; (**iii**) fabrication of plasmonic sensor element via direct laser texturing of the central pillar; (**iv**) deposition of 25-nm thick Au film through a shadow mask. (**b**) Side-view (view angle of 40${}^{\circ }$) scanning electron microscopy (SEM) images of the analyte-enrichment system fabricated on PTFE surface. (**c**,**d**) Close-up SEM images showing details of surface morphology of the hydrophobic pillars and laser-patterned plasmonic site.

We started from direct laser patterning of mechanically polished bulk PTFE substrate, inscribing a rectangular array of 15-μm wide micropillars arranged at a fixed pitch of 95 μm (Figure 1a). At a laser scanning speed of 12.5 mm/s, a pulse energy of 0.85 μJ, and repetition rate 10 kHz, the ablation depth defining the height of the pillars was found to be about 60 μm (see Figure 1b). It should be noted that the applied processing parameters also produced a nanoscale roughness on the pillar sidewalls (see Figure 1b,c), which is explained by re-deposition of the ablated material. This nanoscale roughness, being combined with intrinsic water repellency of the PTFE material, rendered the produced textured surface superhydrophobic with a CA of 170${}^{\circ }$ and CA hysteresis less than 5${}^{\circ }$ [29]. The total size of the patterned surface area was 2×2 mm${}^{2}$, while the fabrication process took ∼20 min and could be boosted by using the galvanometric scanning approach, as tight focusing of the laser beam is not required in this case.

In the center of the laser-processed surface with array of pillars, a larger rectangular-shaped site with the size of 80 × 80 μm${}^{2}$ was left intact (Figure 1a,b). Such a pillar arrangement with a central site coated with superhydrophilic SiO${}_{2}$ was recently shown to work as an efficient analyte-enrichment system [25]. This system supported the Cassie–Baxter state of the water droplet that evaporated on its surface, permitting the deposition of 98% of analyte dissolved in the droplet onto the central site [25]. However, the superhydrophilic SiO${}_{2}$ coating deposited onto the central site, while ensuring efficient analyte concentration, also prevents further utilization of SERS-based methods for ultrasensitive identification of as-deposited analytes, which substantially limits functionality of the proposed device.

To create a fully functional device, in this work we fabricated a SERS-active site combined with the analyte-enrichment system reported in our previous work [25]. To do this, instead of a SiO${}_{2}$ coating we deposited a 600-nm thick Ag film onto the central pillar using the e-beam evaporation procedure performed through the shadow mask (see Figure 1a). The mask protected the other surrounding pillars from metal coverage which would inevitably affect their wetting characteristics and, consequently, the analyte-enrichment performance of the entire device. Next, using a similar fs-laser processing setup, a hexagonal array of spallative textures containing multiple-nanoscale spiky features randomly arranged within circular-shaped craters was produced on the central site surface (Figure 1d). The laser processing parameters applied during this step (focal plane intensity pattern, number of applied pulses, and pulse energy in each pulse) were first tested and optimized on a smooth Ag-coated PTFE to achieve the most pronounced surface relief (see details in the next section). Finally, a 25-nm thick Au film was deposited onto the central pillar above the spallative craters using e-beam evaporation. Noteworthy is that the Au coating plays an important role in optimization of both plasmonic and wetting properties of the central site, which is also discussed in greater detail in the next sections.

### 3.2. Properties of SERS-Active Site and Analyte-Enrichment System

**Figure 2:**
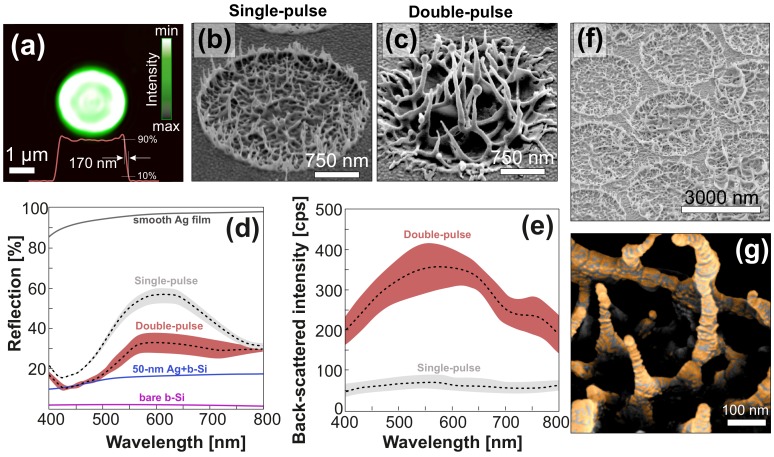
Optimization of plasmonic performance of the sensor element. (**a**) Focal-plane intensity distribution of the flat-top laser beam used to produce spallative textures of the central site. Central cross-sectional intensity profile is also shown in this image by the red curve. (**b**,**c**) Side-view (view angle 40${}^{\circ }$) SEM images of spallative craters produced on the surface of 600-nm thick Ag film under single- and double-pulse laser irradiation. (**d**,**e**) Reflection and dark-field back-scattering spectra measured from the surface area patterned with spallative craters that were produced under single- and double-pulse irradiation. Colored areas near spectra indicate standard deviations from at least 20 spectra measured on different craters. Reflection spectra from a smooth Ag film, as well as from bare and Ag-coated black silicon, are provided as references for comparison on figure (**d**). (**f**) Side-view (view angle 40${}^{\circ }$) SEM image of spallative textures arranged in a hexagonal array with a fixed pitch of 3 μm. (**g**) Close-up false-color SEM image showing non-uniform coverage of isolated nanospikes with Au nanoclusters upon deposition of a 25-nm thick gold film above the spallative craters.

To produce the SERS-active structures on the central site, we chose a rather simple approach based on the spallation (or “swelling”) of a noble (or semi-noble) metal target when subjected to fs laser pulses. The mechanism of this process involves laser-induced melting of a surface layer of the target followed by lift-off of this layer owing to inertial stress confinement [30,31,32,33,34] or even subsurface boiling [35]. As a result, the ejected surface layer leaves a crater of a few microns in diameter and with multiple self-organized nanospikes. Such nanospike-based arrangements were shown to act as efficient plasmon-active nanostructures permitting to enhance photoluminescence and SERS signals from the attached emitters [36,37]. The maximum density of such nanospikes within a single crater (which would ensure the best SERS performance) was previously shown to be tuned by the size of the laser beam (or NA of the focusing lens) [37]. Larger beams were found to provide denser and more uniform nanospikes within the crater [37]. However, utilization of ordinary Gaussian-shaped laser beam focused with a low-NA lens is found to complicate denser arrangement of the spallative textures. This happens because the nanospikes that efficiently absorb incident laser radiation can be eliminated via melting by the beam shoulders upon fabrication of a subsequent crater in a close proximity to the existing one. Therefore, to ensure a dense arrangement of the craters on the central site of the device without smoothing their surface morphology, we used laser projection lithography to generate a flat-top beam with sharp shoulders and a diameter of 2.5 μm (see Figure 2a).

It was also reported that the nanoscale morphology of the craters and their plasmonic properties could be tailored via their irradiation with a subsequent laser pulse with properly adjusted energy E${}_{2}$ [36]. Figure 2b,c reveals how the nanoscale surface morphology evolves under irradiation of the imprinted crater with a second laser pulse at E${}_{2}$ = 240 nJ (the energy of the first pulse was fixed at E${}_{1}$ = 430 nJ, while the time delay between two pulses was 5 ms). Compared with the textures produced by single pulses, the craters produced with double pulses exhibit more pronounced surface morphology with denser arrangements and longer nanospikes. Noteworthy is that given the energy of the first pulse fixed at E${}_{1}$ = 430 nJ, the second pulse only promoted surface morphology when its energy E${}_{2}$ was within the range of 210–270 nJ. Lower pulse energies resulted in smoothening surface morphology through melting of isolated nanospikes, while larger ones led to formation of micro-scale protrusions or a through micro-hole in the center of the crater (not shown here). This defined robust parameter space for irradiation conditions to fabricate pattern of tall “nano-grass”. Along with larger surface-to-volume ratio, the height of the nanospikes is an important factor for light localisation together with their close nanoscale intra-spacing.

The random nanoscale morphology shown in Figure 2b,c makes rigorous electromagnetic simulations of local plasmon-mediated EM fields inaccurate and less informative. To assess and optimize the plasmonic response of such spallative textures, we used a semi-quantitative approach based on correlated measurements of reflection, as well as dark-field back-scattering excited with a properly polarized white light. Typically, a decreased reflectivity indicates the ability of the surface structure to trap the incident radiation, in particular, via coupling with surface plasmons, while the intensity of the scattered light also correlates with the amplitude of the plasmon-mediated EM near-fields [38]. More specifically, when compared with the surface arranged from closely packed textures prepared with single pulses, its counterpart having spallative craters prepared with double pulses is seen in Figure 2d to demonstrate twice lower reflectance over the entire visible spectral range. In comparison with a smooth Ag film, the spallative craters produced with double pulses substantially reduced the average reflection coefficient from 95% to ≈27% (see Figure 2d), indicating good light trapping characteristics of the laser-printed textures. Note that it is usually hard to achieve a strong reduction of the reflection coefficient for highly reflecting metals such as silver, while preserving nanoscale surface roughness required to support visible-light plasmons. To illustrate this feature, in Figure 2d we also provided the reflection spectrum measured from bare and Ag-coated (70-nm thick film) black silicon, the latter being known as a highly performing SERS substrate with outstanding light-trapping characteristics [6,39,40]. The results indicate a substantial increase of the averaged reflection coefficient from 2% to 15% observed upon coating black silicon with a 70-nm thick Ag film. In a similar way, we compared the intensity of DF scattering coming from spallative craters produced with single and double pulses, which can serve as a rough benchmark of the plasmon-mediated EM-field amplitude. Note that for DF measurements we chose an s-polarized white-light pump, which correlates to some extent with an excitation of the spallative crater from the top by linearly polarized source (similar to SERS experiments). This is due to both these pump schemes permitted to predominantly excite oscillations of electron plasma in the direction perpendicular to the surface normal (or nanospikes’ long axis). In comparison with surface areas containing craters produced with single pulse, those produced with double pulses were found to provide an order of magnitude higher intensity of DF scattering, indicating a denser arrangement and higher intensity of electromagnetic hot spots (see Figure 2e). When combined with their reduced reflectivity (which allows more efficient excitation), the strong broadband electromagnetic enhancement supported by the textures printed with double pulses makes these structures promising for SERS-based applications.

The above-mentioned spallative craters with dense hexagonal arrangement were recorded on the central site to combine an SERS-active element with an analyte enrichment system (see Figure 1d and Figure 2f). Further coverage of the central element with a 25-nm thick Au film performed through the shadow mask accomplished the device fabrication process. This last fabrication step played a very important role. More specifically, non-uniform deposition of the Au material above the surface textures provided decoration of Ag nanospikes with Au nanoclusters (see Figure 2e), further boosting plasmonic response of the spallative textures [37]. Additionally, density functional theory calculations recently showed that nanoscale alloying of SERS active metals can support SERS enhancement [41]. Finally, the Au coating rendered the central site hydrophilic. In other words, similar to the previously used SiO${}_{2}$ layer, the Au-coated central site was also able to fix a water droplet on the sensor site without deterioration of its plasmonic properties.

**Figure 3:**
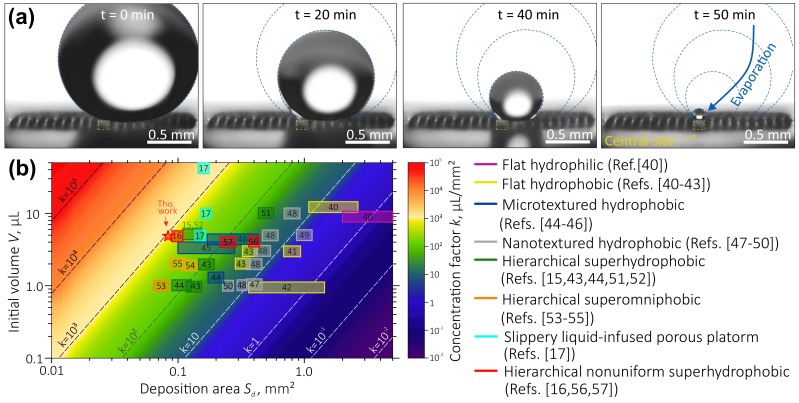
(**a**) Time-lapse optical micrographs illustrating evaporation of a water droplet placed on the device. The position of central site and droplet contours are highlighted by yellow and blue curves, respectively. (**b**) Survey of the concentration factors calculated as a ratio between an initial volume of a water droplet *V* and the deposition area size $S_{d}$ according to previously reported data for the water droplets evaporating on surfaces with different wetting characteristics and morphology including flat hydrophilic [43] and hydrophobic [43,44,45,46] surfaces, microtextured [42,47,48] and nanotextured [49,50,51,52] hydrophobic surfaces, hierarchical superhydrophobic [15,42,46,53,54] and superomniphobic [55,56,57] surfaces, slippery surface [17], as well as hierarchical nonuniform superhydrophobic surfaces [16,58,59]. The result obtained in this work is marked by the red star.

Evaporation of a water droplet (5 μL in volume) placed onto the proposed device is illustrated by a series of time-lapse optical images shown in Figure 3a. These images clearly demonstrate that during drying, the droplet followed the constant-CA mode (characteristic of superhydrophobic surfaces with small values of CA hysteresis [29]), which is consistent with the Cassie–Baxter wetting mode. At the last stage, however, the droplet was found to shift towards the central hydrophylic site, where it finally sticks on its top, even though its initial position was not well aligned with the central site. Such behavior of the drop can be explained in terms of the counterbalancing of the depinning force directed towards the center of the contact area and the pinning force, which is an analog of the friction force [42]. In the case when the pinning force associated with the hydrophilic central site is stronger comparing to those for surrounding superhydrophobic micro-pillars, the center of the contact area between the water droplet and the surface will shift towards central site, as was discussed in detail in [25]. Being strongly stuck to the hydrophilic site, the droplet finally evaporates there, thus concentrating its content atop of the device’s central site. As will be shown below, the proposed analyte enrichment system was able to provide efficient concentration of molecules dissolved in water droplets which were eventually deposited onto the plasmonic-active site with a concentration factor $k=V/S_{d}\approx 800\;\mu$L/mm${}^{2}$ (where *V* is the initial droplet volume and $S_{d}$ is the surface area of central site). In Figure 3b, the concentration factor of the device described in this work is compared with the performance of similar devices reported before. It is clearly seen that the approach presented in this work provides one of the best concentration factors, which is beneficial regarding fabrication cost.

### 3.3. SERS Performance of the Plasmonic Sensor with Analyte Enrichment System

**Figure 4:**
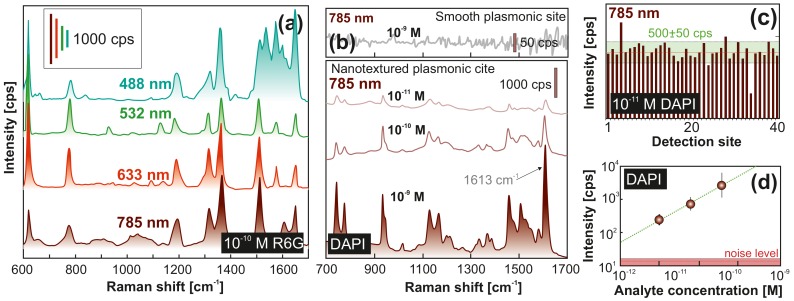
Surface-enhanced Raman scattering (SERS) performance of the fabricated device. (**a**) Series of SERS spectra measured from Rhodamine 6G (R6G) molecules deposited from a 5-μL water drop (initial analyte concentration is 10${}^{-10}$ M) onto the central plasmonic element probed at different pump wavelengths. Corresponding wavelengths of laser source are indicated near each spectrum. (**b**) SERS spectra of 4${}^{\prime }$,6-diamidino-2-phenylindole dihydrochloride (DAPI) molecules dissolved in distilled water at different concentrations and deposited on the sensor element. Representative SERS spectrum of 10${}^{-9}$ M DAPI obtained with a similar sensor with smooth central pillar (i.e., without laser-printed spallative craters) is shown at the top panel (**b**). Each provided SERS spectrum was averaged over 50 similar independent spectra obtained at various detection sites on the central pillar. Fixed pump intensity (≈0.9 mW/μm${}^{2}$) and accumulation time (1 s) were used to obtain all spectra. (**c**) SERS intensity at 1613 cm${}^{-1}$ measured at different detection sites on the central pillar from DAPI molecules deposited from a 5-μL water droplet containing 10${}^{-11}$ M of analyte. The ±10% deviation from the average intensity level is indicated by green-color area. (**d**) SERS intensity at 1613 cm${}^{-1}$ versus DAPI concentration.

To demonstrate the applicability of the proposed device for chemo- and bio-sensing applications, we started from water droplets with Rhodamine 6G (R6G) as analyte. Owing to their large Raman cross-section and good affinity to main plasmon-active metals, R6G molecules are among the most commonly used analytes for proof-of-concept assessment of SERS-active materials. Figure 4a shows SERS spectra of R6G deposited from a 5 μL droplet with its initial concentration of 10${}^{-10}$ M onto the plasmonic element with analyte-enrichment system. We used different pump laser wavelengths ranging from 488 to 785 nm to demonstrate the remarkable performance of the sensor element that exhibited SERS enhancement around 10${}^{7}$–10${}^{8}$ over all the wavelengths used and allowed identification of all main vibration bands of the analyte even at its low initial concentration.

Next, we used the sensor to identify DAPI marker that can strongly bind to adeninethymine-rich regions of DNA, which is why it is often used to stain DNA in histological studies, and bio- and cytochemistry [60]. This analyte does not have specific groups that could absorb on gold, therefore contribution of any chemical enhancement mechanism is expected to be weak for this analyte [9]. Several previous studies reported DAPI detection by means of SERS at concentrations in the range of 10${}^{-4}$–10${}^{-6}$ M [61,62]. Figure 4b provides a series of SERS spectra measured from DAPI probe dissolved in water at concentrations as low as 10${}^{-11}$ to 10${}^{-9}$, indicating the superior performance of the laser-fabricated plasmonic sensor. To illustrate the importance of electromagnetic contribution provided by bimetallic spallative craters to the total SERS yield, we also tested a similar sensor with analyte-enrichment system but with a smooth Ag- and Au-coated central site (without laser-printed textures). For such a device, we did not observe any clear evidence of DAPI Raman bands even at initial DAPI concentration of 10${}^{-9}$ M. Figure 4c gives more statistical information on the performance of the developed device, demonstrating a relatively small variation of the intensity of the main DAPI Raman band at 1613 cm${}^{-1}$ (C=N stretching) measured at different locations on the central pillar. For the probed range of analyte concentrations applied, the averaged intensity of this band plotted against analyte concentration in logarithmic scale (Figure 4d) shows linear behavior. These results substantiate a rather high limit of detection (LoD) above the noise level of the proposed sensor, enabling quantitative identification of analytes that have no chemical affinity to plasmonic materials. Moreover, larger sensitivity also means that faster measurements can be carried out to achieve satisfactory signal-to-noise ratio.

**Figure 5:**
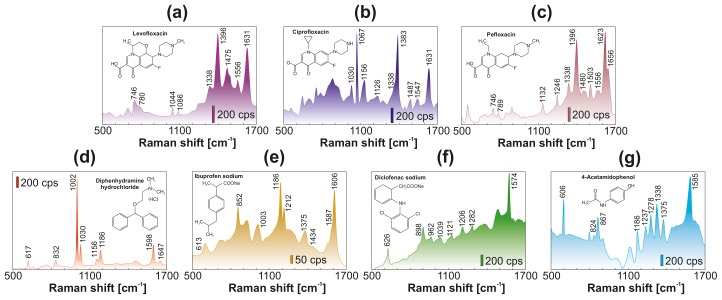
SERS spectra of (**a**) levofloxacin, (**b**) ciprofloxacin, (**c**) pefloxacin, (**d**) diphenhydramine hydrochloride, (**e**) ibuprofen sodium, (**f**) diclofenac sodium, (**g**) 4-acetamidophenol. Each analyte was deposited onto plasmonic site from an evaporating water droplet (5 μL) containing 10${}^{-8}$ M of detected substance. Each spectrum was averaged over at least 30 similar spectra measured at various detection sites. Pump intensity was fixed at 0.9 mW/μm${}^{2}$ and 785 nm as pump wavelength. Signal accumulation time was 1 s for all spectra.

Finally, in recent decades, water quality has become a crucial issue, not only in developing countries, where 90% of sewage is discharged untreated directly into the environment. Due to significant progress in analytical chemistry, previously ignored pollutants were found in food, soil, and even drinking water in the EU and USA, in quantities that pose a serious risk to human health [63,64]. Some of such emerging pollutants, being widely used in medical practice pharmaceuticals and highly toxic chemicals, are of serious concern because of their increased consumption, long-term stability in water and soil, significant effect on the development of bacterial resistance, and poor efficiency of conventional water treatment facilities [65,66,67]. Motivated by this, we tested the newly developed sensing platform with analyte-enrichment system towards SERS-based detection of 7 widely used biologically active organic compounds dissolved in distilled water at 10${}^{-8}$ M concentration: Pefloxacin, Ciprofloxacin, Levofloxacin, Diphenhydramine hydrochloride, Ibuprofen, Diclofenac, and 4-Acetamidophenol. SERS spectra of the tested drugs are shown in Figure 5, indicating the ability of the developed sensor to identify their main characteristic fingerprints (at corresponding concentration of 1.5–3 ppb), which were found to be in good agreement with the spectral position of their Raman bands previously reported for similar analytes [68,69,70,71,72,73,74].

## 4. Conclusions

To conclude, we demonstrated an easy-to-implement laser-printed device for ultrasensitive SERS-based identification of various analytes dissolved in water droplets at trace concentrations. The device combines an analyte enrichment system comprised of properly arranged superhydrophobic micropillars that guide the water droplet with dissolved analyte molecules during its evaporation, so that eventually the concentrated analyte gets deposited onto a hydrophilic central site with SERS-active nanostructures. The proposed device showed remarkable SERS performance, permitting to identify characteristic fingerprints of various model analytes including organic dye molecules and widely used medical drugs at concentration down to 10${}^{-12}$ M in a reliable manner. The high-aspect ratio of nano-structures coated with active nano-alloy of Ag–Au is also promising for gas sensing where volatile organic compounds have to be detected at LoD of 1 ppb or better. This is particularly important for modern energy-efficient zero-emission houses and office buildings which rely on internal air recycling.
